# Using Illusions to Understand Delusions

**DOI:** 10.3389/fpsyg.2012.00407

**Published:** 2012-10-16

**Authors:** Thomas P. White, Sukhi S. Shergill

**Affiliations:** ^1^Department of Psychosis Studies, Institute of Psychiatry, King's College LondonLondon, UK

Clark's article provides a useful synthesis of theoretical and empirical work evincing the brain as a prediction machine and proposing bi-directional, prediction error estimation as a fundamental mechanism of adaptive brain function. Accordingly, in Bayesian terminology both cognitive and perceptual mental states can be understood as statistical posterior distributions formed by integrating encountered data (with specific likelihoods) with prior beliefs (in turn with their own prior distributions). In his discussion of this unifying framework, Clark elegantly describes how updating priors with the aim of minimizing prediction error can sometimes result in illusory experience. When faced with stimuli manipulated to have multiple possible hidden causes, ambiguity is resolved by selecting the most likely explanation given prior beliefs about those hidden causes. As such, in conditions such as those created for the sound-induced visual illusion (Shams et al., 2000), perception is Bayes optimal but illusory. Applying these principles to dysfunctional perception in psychiatric disorders, Clark also evaluates cognitive hypotheses of positive symptoms of schizophrenia, which account for hallucination and delusion as manifestations of disturbances to the system responsible for the generation and weighting of prediction error.

Drawing together these parallel lines of enquiry, this commentary discusses the potential of studying prediction error during illusion-evoking situations in individuals with schizophrenia to improve our understanding of the physiological foundations of the disorder. Schizophrenia is a devastating illness, characterized by the presence of hallucinations (perceptions in the absence of an external stimulus) and delusional beliefs (fixed false beliefs held contrary to evidence); with a marked impact on social and occupational functioning. The underlying mechanisms giving rise to these phenomena remain unclear, and current treatment is largely based on medication to block sub-cortical dopaminergic receptors, allied to cognitive behavioral psychological approaches to reduce distress associated with these symptoms. Examination of individuals with schizophrenia demonstrates that they are less susceptible to perceptual illusions than healthy individuals. For instance, the McGurk phenomenon by which incongruence between certain lip movements and speech sounds produces the perception of alternative speech sounds appears to be exhibited to a lesser degree in individuals with schizophrenia (Pearl et al., 2009). Moreover, these individuals are more proficient at identifying a “hollow” face produced by inverting the binocular depth information of a facial image, than control subjects who illusorily perceive it in its naturally convex state (Schneider et al., 1996, 2002). Dynamic causal modeling of functional magnetic resonance imaging (fMRI) data demonstrates that individuals with schizophrenia exhibit a weakening of top-down processes and a strengthening of bottom-up processes during presentation of “hollow” faces, whereas healthy individuals exhibit a strengthening of top-down processes when encountering the same stimuli (Dima et al., 2009). This finding supports the notion of bi-directional propagation of predictions as discussed by Clark; and recent data support the role of increased top-down control with improved levels of symptoms (Krabbendam et al., 2009). However, there is also data supporting strengthened top-down processes in schizophrenia using the observation of autochthonous delusions, which remain firmly held despite contrary environmental evidence, and also the presence of generalized cognitive biases such as the bias against disconfirmatory evidence (Moritz and Woodward, 2006), suggesting a less transparent role of prediction error estimation in schizophrenia.

Can the findings of reduced susceptibility to perceptual illusions be viewed as conferring a schizophrenia-related behavioral advantage? If so, is this outperformance of healthy individuals by those with schizophrenia best explained by a predictive failure? The findings of our own laboratory provide empirical evidence that predictive abnormalities are not restricted to passive sensation – in accord with Clark's suggestion of prediction as a universal neural mechanism – but extend to predictions made on the basis of self-generated movement. Individuals with schizophrenia differ from healthy individuals in their ability to estimate the level of force necessary to match a force applied directly to themselves (Shergill et al., 2003, 2005). Healthy individuals over-estimate the level of necessary force in this context; implying that accurate prediction of the sensory consequences of an action (by means of a prediction error generated via an efference copy of that action being sent to the sensory system) results in sensory attenuation. The reduction of this effect in schizophrenia despite improved performance indexes relative failure of this system. A similar failure to adequately predict the sensory consequences of inner speech (itself a category of self-action) has been influentially postulated to underlie auditory verbal hallucinations (Frith and Done, 1988), and receives growing neurophysiological support from tasks comparing the auditory processing of self-generated words and the same sounds played back after recording (Ford et al., 2001; Ford and Mathalon, 2005; Simons et al., 2010).

Computational modeling has proven valuable in the characterization of behavioral and neurophysiological parameters relating to the updating of Bayesian processes during information processing in schizophrenia (for example, Averbeck et al., 2011; Evans et al., 2012). Temporal difference algorithms in particular offer an exploitable framework for improving our understanding of the declining influence of old information and integration of new experiences during decision making; their application to perceptual decisions made during illusion-evoking conditions potentially offers an exciting window on the pathophysiology of schizophrenia.

The study of wide-ranging illusions presents a potentially fertile means of understanding the intricacies of predictive failures in schizophrenia as each illusion draws on specific perceptual and cognitive priors. On the flip side, the study of illusion potentially provides a tantalizing model for the study of distorted reality in the healthy brain. Furthermore, given the efficacy of dopaminergic pharmacological neuromodulation in patients with schizophrenia, there is an urgent need for examination of the role of neurotransmitters in mediating predictive coding.
